# A Qualitative Exploration of Self-Management Behaviors and Influencing Factors in Patients With Type 2 Diabetes

**DOI:** 10.3389/fendo.2022.771293

**Published:** 2022-02-17

**Authors:** Xi Peng, Xinhong Guo, Hongmei Li, Dan Wang, Chenxi Liu, Yaling Du

**Affiliations:** ^1^ First Affiliated Hospital, School of Medicine, Shihezi University, Shihezi, China; ^2^ School of Management, Hubei University of Chinese Medicine, Wuhan, China; ^3^ School of Medicine and Health Management, Huazhong University of Science and Technology, Wuhan, China

**Keywords:** qualitative study, diabetes patients, self-care, noncompliance behavior, influencing factors

## Abstract

**Background and Aims:**

The self-management behavior of patients with diabetes involves a complex set of actions involving medication therapy, lifestyle changes, and management of complications in the daily routine. Our study aims to explore adherence to self-management behaviors by patients with type 2 diabetes and the potential factors influencing those behaviors.

**Methods:**

This qualitative study used semi-structured interviews conducted with patients who have type 2 diabetes and who were recruited from the department of endocrinology in a tertiary teaching hospital. Data were analyzed thematically using the interview framework.

**Results:**

Overall, 28 patients with type 2 diabetes were recruited and interviewed. Three types of medication noncompliance behaviors were coded. In particular, blindly optimistic attitudes toward the condition in younger patients who had a short duration of diabetes and fear of or pain from medication therapy were key influencing factors. Irregular monitoring and missed follow-up visits were the most frequently mentioned noncompliance behaviors. Poor understanding of blood glucose monitoring, selective ignorance due to pressure of uncontrolled blood glucose, and blindly optimistic attitudes were also identified as key influencing factors. Dietary behaviors were characterized by an overemphasis on the amount of food in the diet and the preference or declination for particular types of food; ignorance of the dietary structure was present. Misconceptions about dietary and exercise practices were the main types of lifestyles’ noncompliance.

**Conclusion:**

Our study showed the complex picture of noncompliance with self-management behaviors by patients with type 2 diabetes. Noncompliance covered disordered and arbitrary changes in medication therapy, blood glucose monitoring with poorest adherence, lifestyle modifications and complication management. The study findings identify clear challenges to self-management behavior and identify potential key influencing factors. Future interventions and strategies should aim to help patients translate healthcare provider’s information and instructions into action that improve compliance.

## 1 Introduction

Diabetes has become a critical health concern worldwide. The prevalence of type 2 diabetes is rising rapidly in lower- and middle-income countries. In the long term, diabetes is responsible for a substantial death and illness burden. A large national survey showed that an estimated 129.8 million people live with diabetes in mainland China (1–3). The chronic progress of diabetes and its serious complications contribute to substantial socioeconomic and health burdens for individuals and health systems (4).

Self-management is considered as the cornerstone of diabetes management. And self-management behavior has been proven to improve glycemic control and slow the progression or development of complications (5). However, patients with diabetes must follow a complex set of actions involving medication therapy, lifestyle changes, and management of complications in the daily routine (6, 7). Adherence to self-management behaviors is far from satisfactory worldwide (8).

Why do patients with type 2 diabetes fail in adequate self-management practices? Existing studies have investigated the potential barriers associated with self-management in patients with diabetes; these include inadequate information or knowledge about diabetes management and distress or insufficient social support (9–11). Some studies have explored the psychological factors that affect patients with diabetes, such as poor awareness, insufficient self-efficacy, and lack of motivation (12–15).

Focusing on the practice or experience of self-management by patients with diabetes, some qualitative studies have provided insights into self-management behaviors and related dimensions, such as diabetes knowledge, family support, or life circumstances; these studies were conducted in target population in the Netherlands, Pakistan, and other countries (16, 17). The successful application of models or interventions depends not only on the distinctive characteristics of the target population but also on special contexts, such as culture or districts. More exploration of local self-management behaviors, experiences, and key barriers is necessary.

The Chinese health system has run a series of national policies and campaigns aimed at improving self-management in patients with chronic conditions (18); however, suboptimal self-management behaviors are still prevalent (19). To date, several quantitative cross-sectional studies have been conducted in China to measure the status of self-management or explore the relationship between knowledge about diabetes and psychological factors, such as health beliefs or self-efficacy (14, 19, 20). However, limited studies have been conducted to explore in depth the feelings, experiences, and other potential factors related to self-management in patients with type 2 diabetes in China.

Therefore, we developed this qualitative study to explore self-management behaviors and provide insights into the experiences of potential barriers for Chinese patients with type 2 diabetes. The results are key to improving the understanding about and resolving the gaps related to the failure of self-management behaviors by patients with type 2 diabetes, and this better understanding can improve strategies for teaching self-management behaviors to patients with diabetes.

## 2 Materials and Methods

### 2.1 Study Setting

The study was conducted in one tertiary teaching hospital (First Affiliated Hospital of Shihezi University) in the western region of China. In western China, the diabetes prevalence was at a high level of approximately 10.64% to 11.93%, according to the results of one national study (1). With relatively underdeveloped social and economic levels, the western region has few resources for diabetes education (14, 21).

### 2.2 Participants and Sampling

Patients who were diagnosed with type 2 diabetes in the department of endocrinology were recruited for interviews. Participants were admitted to adjust medication therapy for better control of type 2 diabetes or complications and receive professional diabetes education. The participants were asked about their self-management behaviors and especially about deviations from recommended self-management behaviors.

There are two inclusion criteria in participants and sampling section: Patients who were diagnosed with type 2 diabetes in the department of endocrinology were recruited for the interviews. Patients have had a type 2 diabetes diagnosis for at least 1 year, have length of stay over 48 hours, have a willingness to talk about their experiences with the interviewer, and give consent for to be audio recorded. Those who have communication difficulties, such as significant impairment in vision, hearing or speech (assessed by the interviewers) were excluded in the study.

Purposive sampling was applied to ensure that participants with different backgrounds, occupations, and disease durations were included. The participants’ recruitment period lasted from June 1 to July 15 2021.

### 2.3 Interview Framework and Data Collection

The interview framework prepared for the participants concerned self-managements behaviors and their key influencing factors and was based on a literature review. Multiple aspects of self-management—medication therapy, blood glucose monitoring, follow-up visits, and lifestyle changes— were considered. The interviewer collected basic information— age, gender, diabetes duration, and occupation— for each participant. The semi-structured interview framework was presented in **Supplementary File 1**.

### 2.4 Ethics Statement

This study was approved by the ethics committee of the First Affiliated Hospital of Shihezi University Medical School (kj2020-087-03), and it was part of one randomized control trial (Effectiveness of Medication Reconciliation Intervention Led by Clinical Pharmacists Based on Improved Model: a Randomized Controlled Trial), which was registered in the China clinical trial registry (www.chictr.org.cn, number chictr2000035321).

### 2.5 Statistical Analysis

Two authors (D.W. and X.P.) performed the verbatim transcription of the audio recordings of interviews. The interview questions served as the basic framework of analysis. The data were analyzed qualitatively following the steps of content analysis. Two authors (C.X.L and D.W.) separately coded all the interview data line by line using NVIVO software. They compared and classified the codes into similar themes and subthemes. Any discrepancies were discussed with another expert on diabetes management until we agreed on a final list of themes and sub-themes (**Table 1**). No statistical tests were conducted in this study. The qualitative analyses were conducted with NVIVO V.12.1 QSR International software.

**Table 1:** Thematic results of suboptimal self-management behaviors.

Themes	Sub-Theme	No. of Respondents (n=28)
Suboptimal medication therapy behaviors	Discontinuation of medication therapy	10
	Arbitrary changes to medication type, amount, or frequency of therapy	7
	Supplemental health products	2
Suboptimal blood glucose monitoring and follow-up visits behaviors	Lack of regular glucose monitoring	27
	Lack of 2-hour postprandial glucose monitoring	27
	Lack of full-period glucose monitoring	28
	Missed follow-up visits	27
Suboptimal dietary behaviors	Overemphasis of amount of food	10
	Preference or dislike of particular types of food (e.g. preference of coarse grains)	6
Suboptimal exercise behaviors	“Walking as the only way for exercise”	8
	No consideration of activity intensity or consumption of calories	26
Suboptimal behaviors related to managing complications of diabetes	Skincare of diabetic feet	3
	Management of needles for insulin injections	3

## 3 Results

### 3.1 Sample Profile

Overall, 28 patients with type 2 diabetes were recruited to be interviewed. Among the 28 participants, 39% (n=11) were female, and approximately 36% (n=10) were over 60 years old. The youngest participant was a 27-year-old female patient. Diabetes durations ranged from 1 year to more than 30 years, and approximately half of the participants (n=16) lived with diabetes for more than 10 years. Nine patients were diagnosed with type 2 diabetes less than 5 years before the interview. Six patients lived with other chronic conditions, such as hypertension or heart disease. Most patients were retired (n=13), and other occupations included office workers (n=6) or farmers (n=6). The detailed information was presented in **Supplementary File 2**.

### 3.2 Self-Management Behaviors and Key Influencing Factors of Noncompliance Behavior

After identifying the themes and subthemes in this study, the self-management behaviors could be summarized according to the interview framework as medication therapy behaviors, blood glucose monitoring behaviors, dietary and exercise behaviors, and management of disease complications (**Table 1**).

### 3.3 Theme 1: Suboptimal Medication Therapy Behaviors, Trajectories, and Influencing Factors

After the initial diagnosis of type 2 diabetes, patients reported three types of noncompliant behaviors with respect to blood glucose–lowering therapy. There were discontinuation of medication therapy; arbitrary change of frequency, amount, or duration of medication therapy; and use of supplemental health products.

#### 3.3.1 Discontinuation of Medication Therapy

Approximately one-third of the participants (n=10) reported discontinuation of medication, including oral tablets (e.g., metformin or acarbose) and insulin injections.

7 (Male, 51 years with diabetes of 3 years): After my initial diagnosis at the hospital, the doctor gave me two oral tablets. I stopped the medication after several months.

9 (Male, 31 years with diabetes of 3 years): With the initial diagnosis of diabetes with the therapy of insulin injections and oral tablets. After 1 year I stopped all the medication therapy.

#### 3.3.2 Changes to Medication Type, Amount, or Frequency of Therapy

Arbitrary changes in the type, amount, or frequency of the medication made by participants were coded. For example, changes from rapid-acting insulin to long-acting insulin injections or from a glucose-lowering oral tablet to insulin injections were reported.

2 (Female, 78 years with diabetes of 20 years): The doctors recommended I use the rapid-acting insulin three times a day, with the suggestions of diabetic friends, I bought the long-acting insulin for convenience.

28 (Female, 48 years with diabetes of 10 years): When the fasting glucose was higher and cannot reach the targets, I would increase every time….Last time, it was around 20 to 25 units for each meal.

26 (Female, 63 years with diabetes of 2 years): My friends told me that oral tablets would cause renal damage, so I changed the frequency from three times a day to twice a day.

In addition, an arbitrary change to the amount of insulin should be empathized in elderly patients.

#### 3.3.3 Adjustment to Medication Therapy in Elderly Participants

Many participants with a long duration of diabetes conditions told the interviewer that they were familiar with insulin therapy and that they knew how to tackle blood glucose fluctuations. For example, when their measured blood glucose was a little higher or lower than the target, they would increase or decrease therapy by one or two units of insulin.

However, patients, especially elderly patients, did not comprehensively understand insulin therapy, so increased insulin units would increase the risk of hypoglycemia or other severe events, such as ketoacidosis. The following examples demonstrate exaggerated increases in insulin units caused by misunderstandings about medication therapy.

2 (Female, 78 years with diabetes of 20 years): With the diagnosis of hypoglycemia…, when I measured that my blood glucose was higher than the normal times, I increased my insulin to 15 units (The original unit was 12) and try to eat less at that meal. The interviewer: Why were you increasing your insulin at one time and reducing your intake simultaneously, that’s very dangerous. #2: If I did not do that, I cannot lower my blood glucose.

22 (Male, 61 years with diabetes of 14 years): For the last 2 years, I increased my insulin from 16 units to 40 units when my fast blood glucose was not good. Interviewer: How can you increase your insulin in such a large amount? Have you seen a doctor for your insulin treatment? #22: No, I modified the amount by myself. I understood that I can increase my amount, so I increase one or two-unit each time … And it reached 40 units.

#### 3.3.4 Supplemental Health Products

Some patients, especially those who had a relatively long duration of diabetes, referred to traditional Chinese medicine, such as herbal medicine or health products, like bitter vegetables, as a supplementary way to help them lower their blood glucose levels.

3 (Female, 68 years with 15 years of diabetes): I always felt that my blood glucose was not well controlled. I went to the pharmacy. The pharmacy recommended buying the health products. I supplemented my medicine with health products.

10 (Male,49 years with diabetes of 11 years): From 2017, I felt not very good, and my vision weakened. I listened to others’ suggestions to take the traditional way. That was washing my body using herbs, such as ginger.

#### 3.3.5 Trajectory of Suboptimal Medication Therapy Behavior

Two distinctive trajectories of changing medication therapy were coded in this study: a disordered and arbitrary way of changing therapy seen in patients with relatively long durations of diabetes, and an early discontinuation of medication therapy seen in younger patients with short durations of diabetes.

**Figure 1:**
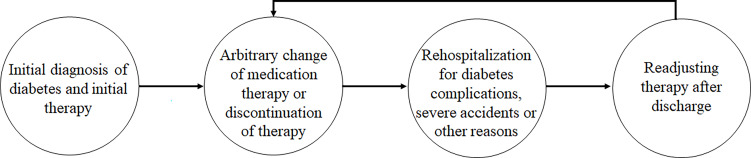
The vicious cycle of medication management of patients with a relatively long duration of type 2 diabetes.

The distinctive feature of a disordered and arbitrary way of changing medication therapy was coded as follows: After the initial diagnosis and medication therapy instruction by physicians, patients arbitrarily changed their medication products, frequency, or form without formal counseling. The first change to their medication therapy varied from several months to several years after the initial diagnosis of diabetes. Rehospitalization was an important opportunity for receiving formal counseling again and for readjusting the medication therapy with a physician. The typical method for formal adjustment of therapy in the hospital and the arbitrary change by patients after discharge are illustrated in **Figure 1**.

4 (Female, 61 years with 20 years of diabetes): After initial diagnosis of diabetes, I did not take any measures. My sister helped me to buy oral glucose-lowering medication…4 to 5 years later, she asked me to go to the hospital to get insulin injections. I used that therapy for around 5 years, and I went to the hospital again for my heart disease. The doctor helped me change my diabetic medication therapy.

22 (Male, 61 years with diabetes of 14 years): After initial diagnosis at the county hospital, I ate the oral tablets by the doctor for around half a month, I changed to another medicine recommended by my diabetic friends. Later after 1 to2 years, I changed to another medication. Then in 2015, I went to the hospital to start my insulin injection……Then I went to the hospital again for my stomachache, and my blood glucose was not good, and the doctor helped me adjust my diabetic therapy.

23 (Male, 53 years with diabetes of 20 years): When I was first diagnosed, I did not take care of it for several years. Then I started oral tablets around 10 years ago. Several years later I went to the hospital to get insulin injections. Then I stopped my oral tablets and increased my insulin units when the glucose was not good.

Patients with a short duration of diabetes (less than 5 years) tended to discontinue their medication therapy at an early stage after the initial diagnosis of diabetes. The time to discontinuation varied from several months to approximately 1 year after the initial diagnosis.

7 (Male, 51 years with diabetes of 3 years): After my initial diagnosis at the hospital, the doctor gave me two oral tablets. I stopped the medication after several months.

9 (Male, 31 years with diabetes of 3 years): With the initial diagnosis of diabetes. I had the therapy of insulin injections and oral tablets. After 1 year I stopped all the medication therapy.

15 (Female, 27 years with diabetes of 2 years): I initially took the metformin and acarbose and insulin injection. However, when I felt the stomachache, I stopped the oral tablets. Later I stopped the insulin injection.

#### 3.3.6 Factors Influencing Noncompliance to Medication Therapy

##### 3.3.6.1 “Feeling Good” as a Reason for Discontinuing Medication by Younger Participants With Diabetes Duration Less Than 5 Years

Participants especially those who were young and had diabetes for a short time said they “*felt good with their conditions*” as the reason for discontinuing their medication therapy. No specific criteria for good control of their conditions were reported. The participants emphasized their subjective feelings or the easing of their symptoms. When they were asked whether their blood glucose levels were assessed, the responses were negative. Youth was mentioned as one reason for arbitrarily discontinuing medication therapy. The young participants thought they could tackle the situation without medication therapy. As a result of their self-perceptions, blood glucose monitoring, regular follow-up visits, and lifestyle modifications were usually abandoned.

7 (Male, 51 years with 3 years of diabetes): When initially diagnosed with diabetes with oral tablets therapy. When I took that therapy for a while, I feel good, so I stopped the medication therapy.

9 (Male, 31 years with diabetes of 3 years): With the initial diagnosis of diabetes with the therapy of insulin injections and oral tablets. After 1 year I felt very good, so I stopped all the medication therapy.

15 (Female, 27 years with diabetes of 2 years): I initially took the metformin and acarbose and insulin injection. However, when I felt the stomachache, I stopped the oral tablets. Later I felt I was Okay … I had no symptoms of thirst or tiredness, I stopped the insulin injection.

21 (Male,47 years and with diabetes of 9 years): The initial treatment therapy 9 years ago was acarbose and I ate for 1 month and stopped therapy. Interviewer: Why you stopped your therapy? For the effects were not good or the cost of the medicine? #21: Not for that reason. I just felt I was young, and It was okay to stop the therapy.

##### 3.3.6.2 Fear of or Pain From Medication Therapy and Roles of Diabetic Family Members and Friends

The participants reported several reasons for noncompliance with medication. Fear or pain from the medication therapy was the most frequently reported barrier to medication adherence. Unsatisfactory glucose-lowering effects, the inconvenience of insulin injections, occupation limitations, and economic burden were also mentioned as reasons for noncompliance.

##### 3.3.6.3 Adverse Effects of Medication Therapy

Most participants chose to change or discontinue medication therapy because of adverse effects from the medication therapy. Examples include stomachache caused by the oral tablets and fear or nervousness caused by the insulin injections.

10 (Male,49 years with diabetes of 11 years): At that time, I took the insulin injections therapy. However, it was painful to take the injections. I was so nervous, so I stopped the insulin.

16 (Female, 76 years with diabetes of 15 years): I felt so hard in my belly, so I discontinued the oral therapy and only took the insulin injections.

17 (Male, 56 years with diabetes of 9 years): The doctor asked me to take insulin injections. When I went home, it felt weird to take the insulin injections and felt like something was pressing me, so I stopped the insulin.

##### 3.3.6.4 Roles of Diabetic Friends and Family Members

Friends and family members with diabetes were important references for changing medication therapy. Participants told interviewers that they trusted and followed the suggestions of friends or family members with diabetes to make changes in the forms, frequencies, or amounts of medication therapy or to supplement therapy with new health products, such as ginger or bitter vegetables, without formal counseling.

4: When I have first diagnosed with diabetes, I did not tackle the problem. And when **my sister** (who was a diabetes patient) asked me to give attention to diabetes and helped me buy the medicine that she used.

22 (Male, 61 years with 14 years of diabetes): With the suggestions of my **diabetic friends**, I bought oral tablets from the pharmacy. Later my diabetic friends told me that oral tablets would cause renal damage, so I went to the hospital to get insulin injections.

10 (Male, 49 years with diabetes of 11 years): When I found that I cannot control my blood glucose well. With the suggestions of **my friends**, I started to “Paojiao” with hot water.

### 3.4 Theme 2:Suboptimal Blood Glucose Monitoring and Follow-Up Behavior and Influencing Factors

#### 3.4.1 Suboptimal Blood Glucose Monitoring

Compared with other dimensions of self-management behavior, participants showed the worst adherence to blood glucose monitoring. Most participants lacked consistent monitoring and regular follow-up visits regardless of the duration of diabetes.

Although almost all the participants expressed that they had bought a blood glucose meter to measure their blood glucose freely at home, only one of them (#13) had the habit of regular fasting blood glucose monitoring. Most patients only measured the fasting blood glucose level; the 2-hour postprandial blood glucose level was rarely measured, despite its importance in guiding medication therapy and lifestyle changes. No participant measured full-period blood glucose levels at home (three fasting glucose measures before a meal, three glucose measures after a meal, and one measure before bedtime).

1 (Male, 59 years and with 10 years of diabetes): Basically, I measured my fasting blood glucose once a week and I rarely measured the 2-hour postprandial blood glucose.

12 (Female, 56 years with diabetes of five years): When I was getting discharged, I usually frequently measured my blood glucose, however, after that period, I feel it was okay and I seldom measured my blood glucose. I measured the fasting blood glucose and postprandial blood glucose was rarely measured.

15 (Female, 27 years with diabetes of 2 years): When I have first diagnosed with diabetes 2 years ago, I never went to the hospital and I never measured my blood glucose.

#### 3.4.2 Follow-Up Behavior

The lack of regular formal follow-up visits was also typical. For some participants, follow-up visits with formal medical counseling were missed for 4 to 5 years. Participant #28 had not attended a follow-up visit for almost 10 years since the initial diagnosis of diabetes. Most participants were rehospitalized after their initial diagnoses because of acute conditions, such as fainting, ketoacidosis, or acute heart failure. Because blood glucose monitoring was inconsistent and follow-up visits were missed, adequate control of blood glucose could not be ascertained.

16: With the initial insulin treatment by the physicians, I did not go to the hospital and adjust the treatment for around 4 to 5 years, I did not come to the hospital because my husband was ill and I had no time to hospital.

17 (Male, 56 years and with diabetes of 9 years): I only had an insulin injection at the hospital and stopped the insulin therapy at discharge. From 2018 on, I never came to the hospital and did not get any tests of my blood glucose. And I came hospital this time because my feet skin gets darker, and I am a little afraid.

25 (Female, 58 years with diabetes of 15 years): I was in another city for around 3 to 4 years so I did not go to the hospital. I came here this time because my feet were rotten.

#### 3.4.3 Factors Influencing Noncompliance With Blood Glucose Monitoring and Follow-Up Visits

##### 3.4.3.1 Lack of Understanding of Blood Glucose Monitoring

Poor understanding about glucose monitoring was the most frequently mentioned contributor for noncompliant behavior. One participant *(#4 Female, 68 years with diabetes of 20 years)* expressed that *“Collecting full period blood glucose in a day was the duty of the hospital, I only need to measure the fasting blood glucose, it was very important”*.

Not knowing the importance of the 2-hour postprandial blood glucose level or not taking the postprandial blood glucose measure seriously was reported as the reason for skipping the postprandial blood glucose measurement.

For example, one participant (Female, 54 years with diabetes of 10 years) said that she did not know about the 2-hour postprandial blood glucose level, despite having diabetes for 10 years and being educated by diabetes coaches in the hospital.

Some participants lacked accurate criteria to target their blood glucose level and felt good about their present blood glucose level. They did not adjust their behavior when they perceived the blood glucose was “good and acceptable”.

9 (Male, 31 years with diabetes of 3 years): After I stopped my insulin, I seldomly measured my blood glucose. It was around 10 to 11 mmol/L and It was only a little bit higher than the normal level. It is Okay.

28 (Female 48 years with diabetes of 10 years): Sometimes, the fasting blood glucose was around 10 mmol/L. It was Okay and acceptable.

##### 3.4.3.2 Stress or Anxiety Related to Uncontrolled Blood Glucose Level

Patients, especially those with poorly controlled diabetes, indicated that they purposely ignored their blood glucose measurements. One participant (Male, 53 years with 20 years of diabetes) expressed that “*I knew that my blood glucose control was poor, and I already measured the fasting blood glucose. The postprandial blood glucose was higher, and I did not want to know the high blood glucose level after meal … it was maybe pressure for me*”.

20 (Male, 66 years with 30 years of diabetes) “Everyone did not like measuring postprandial blood glucose, measuring postprandial blood glucose was not important … And it was difficult for me to insist on measuring fast blood glucose every day, not to mention postprandial blood glucose”.

##### 3.4.3.3 “Feeling Good”

Surprisingly, “feeling good” again was another contributor to noncompliance with blood glucose monitoring in younger participants with short durations of diabetes. Several participants who had a diagnosis less than 5 years stated that they started to reduce the frequency of monitoring the blood glucose and skipped the necessary follow-up when they felt good. They followed the initial therapy without additional monitoring of blood glucose levels or regular follow-up visits (22). Thus, the actual blood glucose level was not measured.

12 (Female, 56 years with diabetes of 5 years): I kept using the initial medication therapy and I felt good. It is Okay When I felt wrong, I would remeasure my blood glucose.

19 (Male, 55 years with diabetes of 5 years): From 2016, I still used the medication therapy 5 years ago and never went to the hospital for follow-up. I feel good so I kept this therapy. Interviewer: What is the meaning of good? Have you measured your blood glucose? #19: It was just the feeling, I did not monitor my blood glucose consistently and only measured when a problem occurred.

##### 3.4.3.4 “No Severe Problems or Accidents Occurred”

With poor knowledge of and awareness about diabetes, the participants perceived that *“diabetes did not threaten their daily life in a long-term way ”*. As long as no severe problems occurred, the patients with relatively long durations of diabetes did not have regular follow-up visits.

23 (Male, 53 years of 20 years of diabetes): Interviewer: Did you know your diabetes was not controlled well? Why did you not come to the hospital? #23: Yes, it was not good. Interviewer: What did you mean good? #23: Good control of diabetes means no severe accidents occurred.

##### 3.4.3.5 Blood Glucose Measurement Method

The method used to measure blood glucose (by collecting fingertip blood) also affected participants’ willingness to measure the full period blood glucose.

16 (Female, 76 years with diabetes of 15 years) “Unlike blood press measuring, measuring the blood glucose hurts. When I went to the community center, I would ask the nurses to help measure my blood glucose, other times I would not”.

### 3.5 Theme 3: Noncompliance With Diet and Influencing Factors

Almost all the participants expressed that they understood the principles of “less eating and more exercise.” The majority of participants said that they modified the dietary aspects in their daily lives, but some patients (n=5) would not change their dietary habits because of occupation or other limitations. The following are examples of patient responses.

9 (Male, 31 years with diabetes of 3 years): As our team ordered the meal together, I had to eat the same food with others. The food the company ordered was always very greasy and not healthy.

14 (Male, 39 years with diabetes of 1 year): When I ate with my coworkers, and I used to follow others’ dietary habits. And I always ate more food than I was alone.

Three major characteristics of dietary behavior were presented as subthems: amount, preference or dislike, and structure of food.

#### 3.5.1 Amount of Food

The amount of the food was overemphasized by the majority of the participants. The participants perceived that “*As long as I eat less, I can lower my blood glucose*.” Several patients expressed confusion, saying that *“I am eating only a bit of the food. What’s wrong with me*? *Why I still cannot control my glucose*?”.

#### 3.5.2 Preference or Dislike of Particular Types of Food

The participants preferred to modify their eating habits by choosing food containing coarse grains or oats, such as corn, oatmeal, beans, which were identified as healthy foods. Sometimes they disliked one particular food, such as meat or the carbohydrate. Meat seemed to become a burden for the patients. Some food (e.g., tomatoes or cucumbers) were considered the best by some patients; they believed these foods contained no calories, they could eat as much of them as they wanted (*#8, Male, 49 years with diabetes of 20 years; #11, Male, 57 years with diabetes of 3 years)*.

#### 3.5.3 Structure of Food

The food structure was rarely mentioned by the participants. The necessary protein would usually be ignored and the match of the nutrition elements, such as carbohydrate, fat, or protein, was not considered.

#### 3.5.4 Influencing Factors on Dietary Noncompliance

The misconceptions about diet contributed to an inappropriate dietary structure for patients with diabetes, resulting in insufficient protein intake or negative events like hypoglycemia. The frequently mentioned misconceptions about diet are listed in **Table 2**. For example, the participants overemphasized the intake of less food as the perceived “right way” to control blood glucose. Participants were overly focused on particular foods they liked or disliked, rather than on nutritional content.

**Table 2:** The main misconceptions according to the characteristics of the dietary noncompliance behavior.

Dietary noncompliance behavior	Examples
**The amount of the food**	*#2: I am afraid to eat too much. I always keep in mind to eat less. Diabetes patients should eat less.* 9 *I did not control my dietary amount before, but now I have to control my amount of food to better control my blood glucose … but I felt a little hungry these months.* 10(Male, 49 years and with diabetes of 11 years). *I reduced half of my dietary intake for lowering my blood glucose*…
**The particular choice of food**	**Preference:** *3(Female, 68 years and with diabetes of 15 years): I like to eat noodles with barley and corns. The barley and corns are good.* *11(Male, 57 years with 3 years of diabetes): I usually would eat steamed buns with elements of coarse grains. It is good for health.* *20: I would like to eat porridge with vegetables. The vegetables in porridge are good.* **Dislike**: 9: *I used not control my amount of intake. Later I took it seriously, and I controlled my intake and especially ate less meat*…… *19: I would eat less meat because I am diabetic. Yesterday I ate vegetables, two steamed buns. I did not eat the meat……*

The specific eating habits during hospitalization was also mentioned by participants. For example, participant #20 reported that “*I was afraid to eat too much at the hospital for fear of blame by the doctors. Maybe I would eat more when I go home. But now I have to control my intake in the hospital*”.

### 3.6 Theme 4: Suboptimal Exercise Behaviors and Influencing Factors

Most participants preferred exercise modifications. However, few patients followed the recommendations of exercise behavior. Most participants older than 60 years old expressed the worries that “*I would feel my knees hurt If I kept walking, so I can only eat less and do no exercise*”.

2: When I was over 60 years old, I cannot do the activity as I was younger. I could only walk slowly for around one hour. And later my knees hurt, and I cannot do the walking, and I had no way to do the exercise.

In patients younger than 60 years without physical limitations, participants indicated that “*I should walk more, that is good for me*”, and only 2 participants mentioned that they considered other types of aerobic exercise or resistance exercise as useful examples of exercise (#12, Female, 56 years with diabetes of 5 years, Table tennis exercise; #14, Male, 39 years of diabetes of 1 year, resistance exercise). No patient mentioned activity indicators, such as activity intensity or consumption of calories.

#### 3.6.1 Occupational Limitations as Influencing Factors on Suboptimal Exercise Behavior

Participants with specific occupations, such as farmers or drivers, or participants with habits of overwork, usually indicated the limitations of exercise behavior modification.

6 (Male, 52 years with diabetes of 11 years): Because I was a farmer, so I had a large number of farming activities in summer and much less activity in the winter. I cannot change the amount of activity.

13: I was very busy with my daily work, and the overtime work usually existed, so I had no time to do the activity.

#### 3.6.2 Misconceptions About Exercise Behavior

Similarly, the frequently mentioned barrier to appropriate exercise, especially in patients older than 60 years, was misunderstanding about exercise goals. Most of the elderly patients understood the need for more exercise and were willing to do the exercise. However, they perceived that *“Walking was the only effective way for exercise, and they could not do the exercise as they were old and their knees would feel pain if they kept walking, so they felt very depressed*”.

Younger patients mentioned, not knowing or understanding that *“the consumption of calories by exercise should be considered, which is related with the intensity, frequency, timing and other medication-related and dietary-related indicators (such as units of insulin, dietary intakes)”* as one reason for their exercise behavior modifications. For example, participant #28 said that: *“I would walk after the meal. I knew that exercise was good for me. Walking was the recommended way. I knew nothing about other aspects of exercise behavior”.*

### 3.7 Theme 5: Behaviors and Influencing Factors Related to Managing Complications of Diabetes

#### 3.7.1 Skincare of Diabetic Feet

Diabetes-related skincare was one risk-reducing behavior ignored by the participants in this study. The participants with diabetic feet mentioned that *“I felt my feet numb and I should take a foot bath ”*. The “Paojiao” (a kind of foot bath) was one traditional way used to relieve the pain and numbness of feet. However, the water temperature and duration of “Paojiao” varied from person to person. The lack of knowledge about skin protection was coded in this study. The participants usually expressed that they did not consider their skin to be fragile, so they just used the “Paojiao” as one good and healthy activity for relieving the numbness of their feet.

17 (Male, 56 years with diabetes of 9 years): I always felt that my feet were numb so I would do the “Paojiao” with hot water. I never considered that the high temperature will hurt my skin.

#### 3.7.2 Management of Needles for Insulin Injections

The replacement of insulin needles was one behavior that deviated from the physicians’ instructions.

3 (Female, 68 years with diabetes of 15 years): When the needles cannot pierce my belly, I would choose to change the needle. I did not know what was the right frequency to replace the needles.

16 (Female, 76 years with diabetes of 15 years): I knew it was the disposal of the needle. But it was a waste if I just used it once…, so I chose to use the needle as long as I can, and sometimes I could feel the needle and it curled in my belly.

## 4 Discussion

This study provides a complex picture of noncompliant self-management behaviors by patients with type 2 diabetes, covering multiple aspects of medication therapy, lifestyle changes, and disease complications.

There is great variability not only in the noncompliant behaviors related to medication therapy but also in the key influencing factors contributing to the noncompliant behaviors.

An arbitrary and disordered way of changing the medication therapy was one emerging theme in patients with a relatively long duration of diabetes. The adverse effects of pain or fear caused by medication therapy and the corresponding references to friends with diabetes have been frequently reported as significant reasons for changing medication therapy (6, 23). A proportion of the participants noted that diabetes did not threaten their daily life, and they only referred to physicians when they were severely ill. An arbitrary change would be rational for them, because they reported that they were familiar with their condition and believed they could handle the situation without formal medical counseling (24). It was likely that a lack of trust in healthcare providers is one potential contributor to nonadherence to medication therapy, as noted in previous studies (25, 26); for example, mistrust and tense relationships exist between physicians and patients in China (27).

In particular, “feeling good” without a formal medical diagnosis was one prominent reason that patients, especially for the younger patients with a short duration of diabetes, discontinued medication therapy. In our study, the younger participants always emphasized that they were young, and they felt good after a short duration of medication therapy. Diagnosis denial, and thus modification of medication use is plausible among young patients (28, 29). One qualitative study showed that, as time progressed and no problems occurred, patients (especially for patients who were diagnosed less than 5 years before) stopped actively managing their diabetes. Patients with a short duration of diabetes, especially younger patients who may have fewer comorbid conditions, would be less susceptible to diabetes complications (30).

It is of great importance for young patients with diabetes to understand the need for self-management as soon as possible. Young adults are developing complications from diabetes in their early 20s, so identifying strategies and interventions to improve self-management in these patients is crucial (31).

Our findings also showed that almost all the participants lacked consistent blood glucose monitoring and regular follow-up visits. Although healthcare providers always emphasize the importance of 2-hour postprandial blood glucose measurement, the majority of patients measured fasting blood glucose and selectively forgot to measure postprandial blood glucose. Such a finding was demonstrated in a previous study, conducted in the middle of China, as well; it showed that, compared with other self-management behaviors, blood glucose monitoring was the behavior with the lowest adherence (19). Poor understanding about how to consistently measure blood glucose each day was the most frequently reported factor leading to missed postprandial blood glucose measurements (19).

However, as one qualitative study indicated, family obligations (caring for family members) and long waiting times (at busy tertiary hospital) were mentioned as the reasons for missing follow-up visits (32). Specifically, younger patients with a short time since diabetes diagnosis showed blindly optimistic attitudes toward their condition, and this optimism is a potential contributor to the absence of regular follow-up visits (24). Without consistent glucose monitoring and regular follow-ups, it is hard for the patients to understand their blood glucose levels and evaluate the efficacy of their medication. Without glucose monitoring and follow-up visits as guidance, the success of an initial medication therapy and the need for adjustment would be unclear.

Regarding lifestyle self-management behaviors, our findings showed that participants understood the principle of “eat less and walk more.” However, they ignored the dietary guidance and instead relied on preferences or dislikes of particular foods. In particular, patients were most interested in coarse grains. Incomplete understanding of appropriate diet choices is one prominent factor contributing to symptoms of poor disease control (including hypoglycemia). In our study, the porridge with barley was not a good choice for patients, because porridge with barley, despite containing healthy grains, is one type of food with a high glycemic index. Such dietary misconceptions have been demonstrated in previous studies; for example, patients with diabetes have perceived a prohibition on carbohydrates (33, 34).

Lack of exercise knowledge was another theme for noncompliance. The misconception that “*Walking was the only way for exercise behavior*” was frequently mentioned, especially by elderly patients. However, alternative types of exercise and important indicators, such as frequency, timing, and intensity of activities and consumption of calories, were rarely mentioned. Professional nutritional and exercise education is necessary for all patients with diabetes, especially because busy physicians seem to have insufficient time to explain the principle of “eat less and walk more” in detail.

Our findings also revealed that skincare was ignored by most patients with the complication of diabetic feet. The traditional Chinese healthy activity “Paojiao” (a kind of foot bath) was one popular activity for relieving the numbness of the diabetic feet. However, “Paojiao” may risk damaging fragile skin, if the water temperature is too high^1^. Management of the needles was another concern, especially in elderly patients. The instructions to change needles regularly seemed wasteful to them; however, the inappropriate use of needles increased the pain or fear of insulin injection and accordingly, negatively affected their adherence to medication therapy.

There were some limitations to this study. First, this study was qualitative and had a small sample with a broad range of disease durations so the results should be interpreted with caution considering the generalizability of the findings. A larger group of patients, or a more homogeneous group should be recruited in future studies. Second, there was potential sample selection bias, because the respondents were all recruited from the same department.

## 5 Conclusions

Our study showed a complex picture of noncompliant self-management behaviors in patients with type 2 diabetes. The disordered and arbitrary changes to medication therapy in patients with a relatively long duration of disease and discontinuation of medication therapy in younger patients with a short duration of diabetes were two distinctive suboptimal medication behavior. Blood glucose monitoring and regular follow-up visits were the behaviors with poorest compliance. Lack of knowledge and misconception of dietary and exercise practices were the contributors to improper lifestyle modification. Future interventions and strategies can be implemented according to these observed self-management behaviors and their key influencing factors.

## Data Availability Statement

The raw data supporting the conclusions of this article will be made available by the authors, without undue reservation.

## Ethics Statement

The studies involving human participants were reviewed and approved by the First Affiliated Hospital of Shihezi University Medical School (kj2020-087-03). Written informed consent for participation was not required for this study in accordance with the national legislation and the institutional requirements.

## Author Contributions

DW and XP helped design the study. XP and XG performed the data collection and initial analyses and drafted the initial manuscript. XP, HL, and YD helped design the study, reviewed and revised the manuscript. DW, XP and CL helped reviewed and revised the manuscript. All authors contributed to the article and approved the submitted version.

## Funding

This study was supported by The National Natural Science Fund Program “Communication preference and patterns with patients with chronic diseases based on best-worst scaling and latent class analysis” (Grant Number: 72004066).

## Conflict of Interest

The authors declare that the research was conducted in the absence of any commercial or financial relationships that could be construed as a potential conflict of interest.

## Publisher’s Note

All claims expressed in this article are solely those of the authors and do not necessarily represent those of their affiliated organizations, or those of the publisher, the editors and the reviewers. Any product that may be evaluated in this article, or claim that may be made by its manufacturer, is not guaranteed or endorsed by the publisher.

## Footnotes

1
https://www.diabetes.org/diabetes/complications/skin-complications
