# Supplementary material for: A Qualitative Exploration of Self-Management Behaviors and Influencing Factors in Patients With Type 2 Diabetes
Source: Front Endocrinol (Lausanne). 2022 Feb 17;13:771293. doi: 10.3389/fendo.2022.771293 (PMC8893955; doi:10.3389/fendo.2022.771293)
Supplement: Supplementary file 1 [file DataSheet_1.pdf]

Supplementary file 1 Interview Framework

Introductory question

Let's talk about your diabetes, and how and when you were first diagnosed with diabetes?

Noncompliance self-management behaviors and influencing factors

Medication therapy

- What was your initial therapy recommended by the physician?
- Did you follow the initial therapy?
- How did you take your medication?
- When did you change your medication therapy? (Discontinue/Forms/Amount/Frequency)
- Why did you make the changes? (Side effects/Forget/Financial burden/Busy/Work limitations)

Monitoring of blood glucose

- Did you have blood glucose monitoring meter at home?
- Did you monitor your blood glucose level? (fast blood glucose or 2-hour postprandial blood glucose or both?)
- What was the frequency of monitoring blood glucose?
- How did you handle blood glucose fluctuation? (Go to the physician/Increase or decrease the amount of insulin/Ignorance)
- When did you change your monitoring behavior and Why did you make the changes (Economic burden/Afraid of needles/Psychological factor)?

Follow-up visits

- Did you go to have a follow-up visits when you were initially diagnosed with diabetes?
- What was the frequency of follow-up visits?
- Why did you not have regular follow-up visits?

Dietary behaviors

- What dietary modification recommend to you by physicians?
- Did you follow your dietary modification?
- When did you change your dietary behavior and why did you make the changes? (Work limitations/Personal likes or dislikes)
- Please tell me your current diet. (Amount/Type/Structure)

Exercise behaviors

- What type of exercise was recommended to you by physicians?
- Did you follow the exercise modification?
- When did you change your exercise behavior and why did you make the changes?
- Please tell me your current exercise behavior. (Frequency/Intensity)
