# Supplementary material for: A Qualitative Exploration of Self-Management Behaviors and Influencing Factors in Patients With Type 2 Diabetes
Source: Front Endocrinol (Lausanne). 2022 Feb 17;13:771293. doi: 10.3389/fendo.2022.771293 (PMC8893955; doi:10.3389/fendo.2022.771293)
Supplement: Supplementary file 2 [file DataSheet_2.pdf]

Supplementary file 2 Demographic characteristics of the participants (n=28)

Respondent	Age	Gender (1 Male/ 2 Female)	Duration of diabetes (years)	Occupation (1 Retired/2 Office worker)/3 Farmer/4 Driver/5 Other types
#1	59	1	10	5
#2	78	2	20	1
#3	68	2	15	1
#4	61	2	20	1
#5	77	1	20	1
#6	52	1	11	3
#7	51	1	3	2
#8	49	1	20	3
#9	31	1	3	2
#10	49	1	11	3
#11	57	1	3	1
#12	56	2	5	2
#13	39	1	4	2
#14	39	1	1	5
#15	27	2	2	2
#16	76	2	15	1
#17	56	1	9	4
#18	64	1	6	1
#19	55	1	5	3
#20	66	1	30	1
#21	47	1	9	3
#22	61	1	14	1

#23	53	1	20	3
#24	54	2	10	1
#25	58	2	15	1
#26	63	2	2	1
#27	78	2	30	1
#28	48	2	10	2
